# Indications and Outcomes of Non-Emergent Cardiac Surgery Following Transcatheter Aortic Valve Replacement

**DOI:** 10.1093/icvts/ivag142

**Published:** 2026-06-02

**Authors:** Younus Qamar, Hartzell V Schaff, Kevin L Greason, Gabor Bagameri, Joseph A Dearani, Kimberly A Holst, Alberto Pochettino, Arman Arghami, Austin Todd, Juan A Crestanello

**Affiliations:** Department of Cardiovascular Surgery, Mayo Clinic, Rochester, MN 55905, United States; Department of Cardiovascular Surgery, Mayo Clinic, Rochester, MN 55905, United States; Department of Cardiovascular Surgery, Mayo Clinic, Rochester, MN 55905, United States; Department of Cardiovascular Surgery, Mayo Clinic, Rochester, MN 55905, United States; Department of Cardiovascular Surgery, Mayo Clinic, Rochester, MN 55905, United States; Department of Cardiovascular Surgery, Mayo Clinic, Rochester, MN 55905, United States; Department of Cardiovascular Surgery, Mayo Clinic, Rochester, MN 55905, United States; Department of Cardiovascular Surgery, Mayo Clinic, Rochester, MN 55905, United States; Department of Quantitative Research Methods, Mayo Clinic, Rochester, MN 55905, United States; Department of Cardiovascular Surgery, Mayo Clinic, Rochester, MN 55905, United States

**Keywords:** transcatheter aortic valve replacement, structural valve deterioration, non-structural valve deterioration, prosthetic valve endocarditis, structural valve disease

## Abstract

**Objectives:**

This study examined the indications, frequency, and outcomes of cardiac surgery following transcatheter aortic valve replacement (TAVR) at a single institution. As TAVR volumes increase, understanding the nature and outcomes of subsequent cardiac operations is critical, particularly as the procedure expands to younger, lower-risk populations.

**Methods:**

We analysed outcomes of 61 patients who underwent cardiac surgery after TAVR at our institution from August 2011 to September 2023, excluding periprocedural complications and staged procedures. Patients were stratified into 2 groups: those requiring surgical aortic valve replacement (SAVR) with or without concomitant procedures (*n* = 33) and those undergoing non-SAVR cardiac operations (*n* = 28). Data were collected from a prospectively maintained cardiovascular surgery database and electronic health records. Indications for surgery, operative characteristics, and outcomes were analysed, with survival assessed using Kaplan-Meier estimates.

**Results:**

The median interval between TAVR and cardiac surgery was 19 months. Indications for SAVR included infective endocarditis (36%), non-structural valve deterioration (36%), structural valve deterioration (12%), and valve thrombosis (6%). Non-SAVR operations primarily addressed mitral valve disease (43%) and coronary artery disease (29%). Operative mortality was 6.6%, with no deaths in the endocarditis subgroup. Postoperative complications included prolonged mechanical ventilation (18%), new-onset renal failure (7%), and stroke (2%). Kaplan-Meier survival estimates were 83% at 1 year and 50% at 5 years.

**Conclusions:**

Although complex, cardiac operations in patients who have undergone TAVR can be performed with acceptable mortality rates. Structural valve deterioration, paravalvular leak, and endocarditis were the primary indications for SAVR, while mitral valve and coronary artery disease predominated in non-SAVR cases. These findings highlight the importance of considering nonaortic valve pathologies in TAVR planning, particularly as the procedure expands to younger, lower-risk populations.

## Graphical abstract

**Figure:**
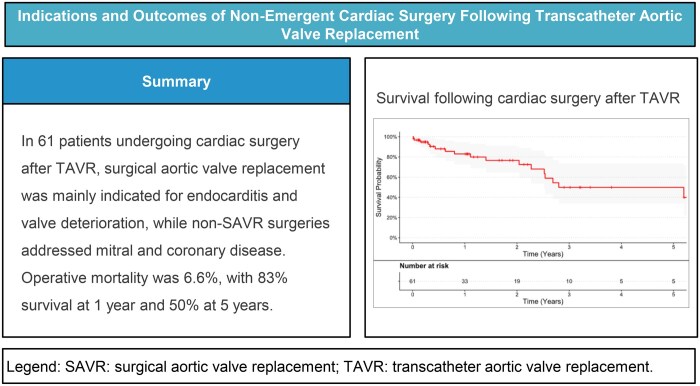

## INTRODUCTION

In 2011, transcatheter aortic valve replacement (TAVR) was approved by the United States Food and Drug Administration for severe aortic stenosis in patients with high or prohibitive surgical risk.^1^ Its subsequent approval for use in low and intermediate-surgical-risk patients significantly increased TAVR volumes worldwide.^2^^,^^3^ As a result, an increasing number of patients now require cardiac surgery after TAVR.^4–11^ However, there are few reports detailing the indications for and outcomes of these operations. Therefore, we aimed to examine the indications, frequency, and outcomes of cardiac surgery after TAVR at our Clinic.

## METHODS

### Study population

This study was approved by the Institutional Review Board at Mayo Clinic, Rochester, Minnesota (IRB No: 23-011420) on December 6, 2023. The informed consent requirement was waived because of the retrospective nature of the study. We identified all consecutive patients who underwent cardiac surgery following TAVR at our institution from August 2011 to September 2023. Patients who required emergent cardiac surgery to manage periprocedural complications (ie, within 30 days of the TAVR procedure) were excluded. Further, patients who underwent planned cardiac surgery as a staged procedure following TAVR, including off-pump coronary artery bypass grafting (CABG) and pericardiectomy, were also excluded.

### Data collection

Data were retrieved from our institution’s prospectively maintained cardiovascular surgery database and patients’ electronic health records. The Society of Thoracic Surgeons-Predicted Risk of Mortality (STS-PROM) was used to predict mortality risk at the time of the initial TAVR procedure and the time of subsequent cardiac surgery. TAVR bioprosthetic valve dysfunction was categorized by the Valve Academic Research Consortium definitions as structural valve dysfunction, non-structural valve dysfunction, thrombosis, or endocarditis.^12^^,^^13^ Vital status was ascertained using information from the Mayo Clinic registration database and LexisNexis Accurint (New York City, NY), which collates information from multiple sources, including the Social Security Death Master File and state health records.

### Statistical analysis

Categorical variables are expressed as frequencies and percentages, and continuous variables are expressed as medians and IQRs. Patients were stratified into 2 groups for descriptive analyses: (1) those who underwent SAVR with or without concomitant cardiac procedures and (2) those who underwent non-SAVR cardiac procedures following a previous TAVR procedure. Differences between the 2 study groups were assessed using the Wilcoxon rank-sum test for continuous variables, and either the Pearson *χ*^2^ or Fisher exact test for categorical variables. The Kaplan-Meier method was used for survival analysis. All statistical analyses were performed using R statistical software (version 2.2; R Foundation for Statistical Computing).

## RESULTS

### Patient characteristics

During the study period, 61 patients underwent cardiac surgery at a median interval of 19 months (IQR, 11-33 months) after an initial TAVR (**Table 1**). Patients were predominantly male (54%) with a median age of 73 years (IQR, 68-79 years; range, 36-89 years) at the time of their cardiac surgery. Hypertension, dyslipidaemia, and coronary artery disease were the most common associated comorbidities. At the time of surgery, 44 (77%) patients had NYHA class III or IV symptoms. The median STS-PROM, available for 29 (48%) patients, was 5.6% (IQR, 3.2%-12.7%), including 18 (29%) patients who were categorized as low- or intermediate-risk (STS-PROM <8%).

**Table 1.** Baseline Clinical Characteristics of the Study Cohort

	Total (*N* = 61)	Non-SAVR (*N* = 28)	SAVR after TAVR (*N* = 33)	*P*-value
Demographics				
Age, years	72.8 (68.2, 79.2)	73.2 (69.6, 79.9)	71.1 (68.1, 76.9)	.377
Female sex	28 (45.9)	16 (57.1)	12 (36.4)	.105
Body surface area, m^2^	2.0 (1.8, 2.1)	2.0 (1.8, 2.1)	2.0 (1.9, 2.2)	.304
Comorbidities				
Atrial fibrillation/flutter	16 (26.2)	10 (35.7)	6 (18.2)	.121
Cerebrovascular disease	18 (29.5)	7 (25.0)	11 (33.3)	.477
Chronic lung disease	19 (31.1)	8 (28.6)	11 (33.3)	.689
Coronary artery disease	33 (54.1)	17 (60.7)	16 (48.5)	.340
Diabetes mellitus	25 (41.0)	10 (35.7)	15 (45.5)	.441
Dialysis	3 (4.9)	1 (3.6)	2 (6.1)	.654
Dyslipidaemia	55 (90.2)	25 (89.3)	30 (90.9)	.832
Hypertension	59 (96.7)	27 (96.4)	32 (97.0)	.906
Peripheral vascular disease	20 (32.8)	12 (42.9)	8 (24.2)	.123
Renal failure	9 (14.8)	5 (17.9)	4 (12.1)	.529
Preoperative PPM and/or ICD	20 (32.8)	9 (32.1)	11 (33.3)	.921
Mediastinal irradiation	7 (11.5)	7 (25.0)	0 (0.0)	<.001
NYHA class III or IV symptoms	44 (72.1)	23 (82.2)	21 (63.6)	.274
Previous cardiac surgery				
Any previous cardiac surgery	29 (47.5)	13 (46.4)	16 (48.5)	.873
Aortic surgery	6 (9.8)	2 (7.1)	4 (12.1)	.515
Coronary artery bypass grafting	15 (24.6)	5 (17.9)	10 (30.3)	.261
Mitral valve repair	3 (4.9)	2 (7.1)	1 (3.0)	.459
Mitral valve replacement	2 (3.3)	1 (3.6)	1 (3.0)	.906
Pericardiectomy	2 (3.3)	1 (3.6)	1 (3.0)	.906
Surgical aortic valve replacement	21 (34.4)	9 (32.1)	12 (36.4)	.730
Tricuspid valve repair	2 (3.3)	2 (7.1)	0 (0.0)	.118
Tricuspid valve replacement	20 (32.8)	9 (32.1)	11 (33.3)	.921
TAVR valve in-situ				.384
Balloon-expandable valve	47 (77.0)	23 (82.1)	24 (72.7)	
Self-expandable valve	14 (23.0)	5 (17.9)	9 (27.3)	
Interval between TAVR and subsequent cardiac surgery, months	19.0 (11.0, 33.0)	27.0 (11.8, 38.2)	16.0 (11.0, 28.0)	.238

Data are expressed as median (first quartile, third quartile) or as number (percentage).

Abbreviations: ICD = implantable cardioverter defibrillator; NYHA = New York Heart Association; PPM = permanent pacemaker; SAVR = surgical aortic valve replacement; TAVR = transcatheter aortic valve replacement.

Of the 61 patients, 36 (59%) had undergone TAVR at the Mayo Clinic. The median pre-TAVR STS-PROM, available for 40 patients (primarily those who had their TAVR procedure at the Mayo Clinic), was 3.9% (IQR, 2.2-6.9). A balloon-expandable TAVR valve (Edwards Lifesciences Sapien valve) was implanted in 47 (77%), while 14 (23%) received a self-expandable valve (Medtronic CoreValve). Twenty-nine (48%) patients had previously undergone cardiac surgery prior to their TAVR procedure, including 10 with prior SAVR (16% of the cohort), 15 with prior CABG (25%), and 6 with prior aortic surgery (10%).

### Indications for cardiac surgery after TAVR

In the 2 subcohorts, 28 patients underwent non-SAVR cardiac surgery after TAVR, and 33 (54%) underwent SAVR (with or without concomitant cardiac procedures) after TAVR. Redo SAVR was performed due to infective endocarditis in 12 (36%) patients, non-structural TAVR valve deterioration in 12 (36%) patients, structural TAVR valve deterioration in 4 (12%) patients, and TAVR valve thrombosis in 2 (6%) patients (**Table 2**). Non-SAVR cardiac surgery was performed due to progression in mitral or tricuspid valve diseases, as well as coronary artery disease. Baseline clinical characteristics were similar between the 2 subcohorts.

**Table 2.** Indication for Cardiac Surgery after TAVR

Indications	No. (%)
TAVR valve endocarditis	12 (19.7)
TAVR valve thrombosis	2 (3.3)
Structural TAVR valve deterioration	4 (6.6)
Non-structural TAVR valve deterioration	12 (19.7)
Mitral valve stenosis/regurgitation	13 (21.3)
Tricuspid valve regurgitation	8 (13.1)
Pulmonary valve regurgitation	1 (1.6)
Subacute type A aortic dissection	2 (3.3)
Coronary artery disease	10 (16.4)
End-stage heart failure (dilated cardiomyopathy)	2 (3.3)
Other indications	5 (8.2)

### Preoperative echocardiographic findings

As seen in **Table 3**, the median left ventricular ejection fraction (LVEF) was normal (60%; IQR, 55%-64%), and the median pulmonary artery systolic pressure (PASP) was 38.5 mmHg (IQR, 30.8-51.2 mmHg). As expected, patients who underwent SAVR after TAVR were more likely to have prosthetic aortic valve stenosis (39% vs 0%; *P* < .001), and/or aortic valve regurgitation (27% vs 7%; *P* = .002) compared to those who underwent non-SAVR cardiac surgery.

**Table 3.** Preoperative Echocardiographic Measurements

	Total (*N* = 61)	Non-SAVR (*N* = 28)	SAVR after TAVR (*N* = 33)	*P*-value
LV ejection fraction, %	60 (55, 64)	60 (55, 63)	60 (55, 64)	.606
≤30%	3 (4.9)	2 (7.1)	1 (3.0)	
PASP, mmHg	38.5 (30.8-51.2)	39.0 (32.0-46.0)	38.0 (29.0-52.5)	.805
≥40 mmHg	23 (37.7)	11 (39.3)	12 (36.4)	
Moderate or severe				
Aortic regurgitation	11 (18.1)	2 (7.1)	9 (27.3)	.005
Aortic stenosis	13 (21.3)	0 (0.0)	13 (39.4)	<.001
Mitral regurgitation	20 (32.8)	10 (25.7)	10 (30.3)	.032
Mitral stenosis	10 (16.4)	7 (25.0)	3 (9.1)	.202
Tricuspid regurgitation	23 (37.7)	14 (50.0)	9 (27.3)	.334
Pulmonary regurgitation	2 (3.3)	2 (7.1)	0 (0.0)	.049

Data are expressed as median (first quartile, third quartile) or as number (percentage).

Abbreviations: LV = left ventricular; PASP = pulmonary artery systolic pressure; SAVR = surgical aortic valve replacement; TAVR = transcatheter aortic valve replacement.

### Operative characteristics

A median sternotomy approach was adopted for all but 1 patient, who received a posterolateral thoracotomy incision for placement of a left atrial-to-left ventricle valved conduit to bypass a heavily calcified and stenotic mitral valve (MV); in the overall cohort, 44% were multiple redo sternotomies and 39% were categorized as urgent procedures (**Table 4**). The median cardiopulmonary bypass (CPB) time was 125 min (IQR, 91-120 min), and the median aortic cross-clamp time was 98 min (IQR, 60-173 min). Circulatory arrest was utilized in 7 patients with a median arrest time of 21 min (IQR, 20-26 min).

**Table 4.** Operative Characteristics

	Total (*N* = 61)	Non-SAVR (*N* = 28)	SAVR after TAVR (*N* = 33)	*P*-value
Status				.289
Elective	37 (60.7)	19 (67.9)	18 (54.5)	
Urgent	24 (39.3)	9 (32.1)	15 (45.5)	
Sternotomy				.548
Primary	33 (54.1)	15 (53.6)	18 (54.5)	
Secondary or more	27 (44.3)	11 (39.3)	16 (48.5)	
Operative times, mins				
CPB time	125 (91, 210)	84 (63, 118)	172 (125, 250)	<.001
Aortic cross-clamp time	98 (60, 173)	53 (44, 87)	148 (98, 205)	<.001
Circulatory arrest time	21 (20, 26)^a^	25 (22, 28)^b^	21 (20, 21)^c^	1.000
Perioperative mechanical circulatory support				
Intra-aortic balloon pump	2 (3.3)	1 (3.6)	1 (3.0)	.906
Extracorporeal membrane oxygenation	1 (1.6)	0 (0.0)	1 (3.0)	.353
Cardiac surgery performed				
Coronary artery bypass grafting	12 (19.7)	8 (28.6)	4 (12.1)	.107
SAVR with aortic root replacement	11 (18.0)	0 (0.0)	11 (33.3)	<.001
SAVR without aortic root replacement	22 (36.1)	0 (0.0)	22 (66.7)	<.001
Ascending aorta replacement	15 (24.6)	2 (7.1)	13 (39.4)	.004
Mitral valve replacement	12 (19.7)	9 (32.1)	3 (9.1)	.024
Mitral valve repair	6 (9.8)	3 (10.7)	3 (9.1)	.832
Tricuspid valve replacement	6 (9.8)	6 (21.4)	0 (0.0)	.005
Tricuspid valve repair	4 (6.6)	1 (3.6)	3 (9.1)	.385
Septal myectomy	2 (3.3)	1 (3.6)	1 (3.0)	.906
Pericardiectomy	2 (3.3)	2 (7.1)	0 (0.0)	.118
Orthotopic heart transplant	3 (3.3)	3 (7.1)	1 (0.0)	.118

Data are expressed as median (first quartile, third quartile) or as number (percentage).

a *n* = 7.

b *n* = 2.

c *n* = 5.

Abbreviations: CPB = cardiopulmonary bypass; SAVR = surgical aortic valve replacement; TAVR = transcatheter aortic valve replacement.

The surgical procedure undertaken varied by patient cohort. Among those undergoing TAVR explant, a concomitant aortic enlargement or aortic root procedure was necessary during valve replacement in 64% (31% root/annular enlargement, 33% root replacement). Notably, the need for aortic root enlargement or replacement was similar between self-expandable and balloon-expandable TAVRs (36% vs 33%). In this cohort, CABG was performed concomitantly in 12%, with MV repair or replacement in 18%. Among those undergoing non-SAVR cardiac surgery after TAVR, the most common procedures were MV repair or replacement (43%), CABG (29%), and tricuspid valve repair or replacement (25%). Importantly, 2 (7%) patients in this cohort required non-SAVR aortic procedures. Further, among patients who underwent non-SAVR cardiac surgery, 2 (7%) required circulatory arrest for ascending aorta replacement. In contrast, 5 (15%) patients who underwent TAVR explant followed by SAVR required circulatory arrest, including 4 (12%) for hemiarch replacement and 1 (3%) for total arch replacement. Reflective of the higher prevalence of complex aortic surgery, the median increase in CPB and aortic cross-clamp times compared with the non-SAVR group was 88 and 95 min, respectively.

### Prosthetic TAVR valve endocarditis

At a median interval of 19.5 months (range: 9.8-35.8 months) from the initial TAVR, 12 (20%) patients in the overall cohort required reintervention for TAVR valve endocarditis; 9 of these cases were categorized as urgent procedures. Of the 12 infective TAVRs, 10 were balloon-expandable valves, and 2 were self-expandable valves. Of the 10 patients with infected balloon-expandable valves, 9 received a mechanical prosthesis, including 6 patients who underwent concomitant aortic root replacement. Similarly, of the 2 patients with infected self-expandable valves, 1 received a mechanical prosthesis with concomitant aortic root replacement, while the other underwent an isolated aortic valve replacement with a bioprosthesis. Importantly, no in-hospital deaths occurred within this subcohort of 12 patients with prosthetic TAVR valve endocarditis.

### Postoperative complications and early outcomes

Operative mortality was 6.6%. Causes of death included multisystem organ failure in 2 patients, mesenteric ischaemia in 1 patient, and cardiogenic shock in 1 patient. Operative complications, summarized in **Table 5**, were generally similar between patients who underwent non-SAVR cardiac surgery and those who underwent SAVR after TAVR. Mechanical circulatory support was required in 3 (5%) patients. Median length of stay in ICU was 84.8 hours (IQR, 42.8-162.8 hours), and the median length of stay in hospital was 10 days (IQR, 6-15 days).

**Table 5.** Postoperative Complications and Early Outcomes

	Total (*N* = 61)	Non-SAVR (*N* = 28)	SAVR after TAVR (*N* = 33)	*P*-value
Postoperative complications				
Blood transfusion	46 (75.4)	23 (82.1)	23 (69.7)	.261
Atrial fibrillation	18 (29.5)	10 (35.7)	8 (24.2)	.328
Prolonged mechanical ventilation (>24 hours)	11 (18.0)	6 (21.4)	5 (15.2)	.525
Permanent pacemaker	6 (9.8)	1 (3.6)	5 (15.2)	.130
New-onset renal failure requiring dialysis	4 (6.6)	2 (7.1)	2 (6.1)	.865
Deep sternal wound infection	2 (3.3)	2 (7.1)	0 (0.0)	.118
Stroke	1 (1.6)	0 (0.0)	1 (3.0)	.353
Re-exploration for bleeding	5 (8.2)	3 (10.7)	2 (6.1)	.509
Reoperation for valve dysfunction	1 (1.6)	0 (0.0)	1 (3.0)	.353
Operative mortality	4 (6.6)	3 (10.7)	1 (3.0)	.231
Length of stay				
Intensive care unit, hours	84.8 (42.8, 162.8)	107.3 (46.6, 164.2)	64.1 (39.5, 158.0)	.385
Postoperative, days	10 (6, 15)	12 (7, 21)	9 (6, 12)	.220
Readmission within 30 days of surgery	5 (8.2)	3 (10.7)	2 (6.1)	.513

Data are expressed as median (first quartile, third quartile) or as number (percentage).

Abbreviations: SAVR = surgical aortic valve replacement; TAVR = transcatheter aortic valve replacement.

Postoperative atrial fibrillation occurred in 18 (30%) patients, and 11 (18%) required prolonged mechanical ventilation. New-onset renal failure requiring dialysis was reported in 4 (7%) patients, and the incidence of postoperative stroke was 2%. Although not statistically significant, 15% of patients who underwent SAVR after TAVR required a permanent pacemaker compared to 4% of patients who underwent non-SAVR cardiac surgery (*P* = .205). Five (8%) of patients underwent re-exploration for bleeding, and 1 (2%) patient required reoperation for valve dysfunction. Five (8%) patients were readmitted within 30 days of surgery.

### Midterm survival

**Figure 1.**
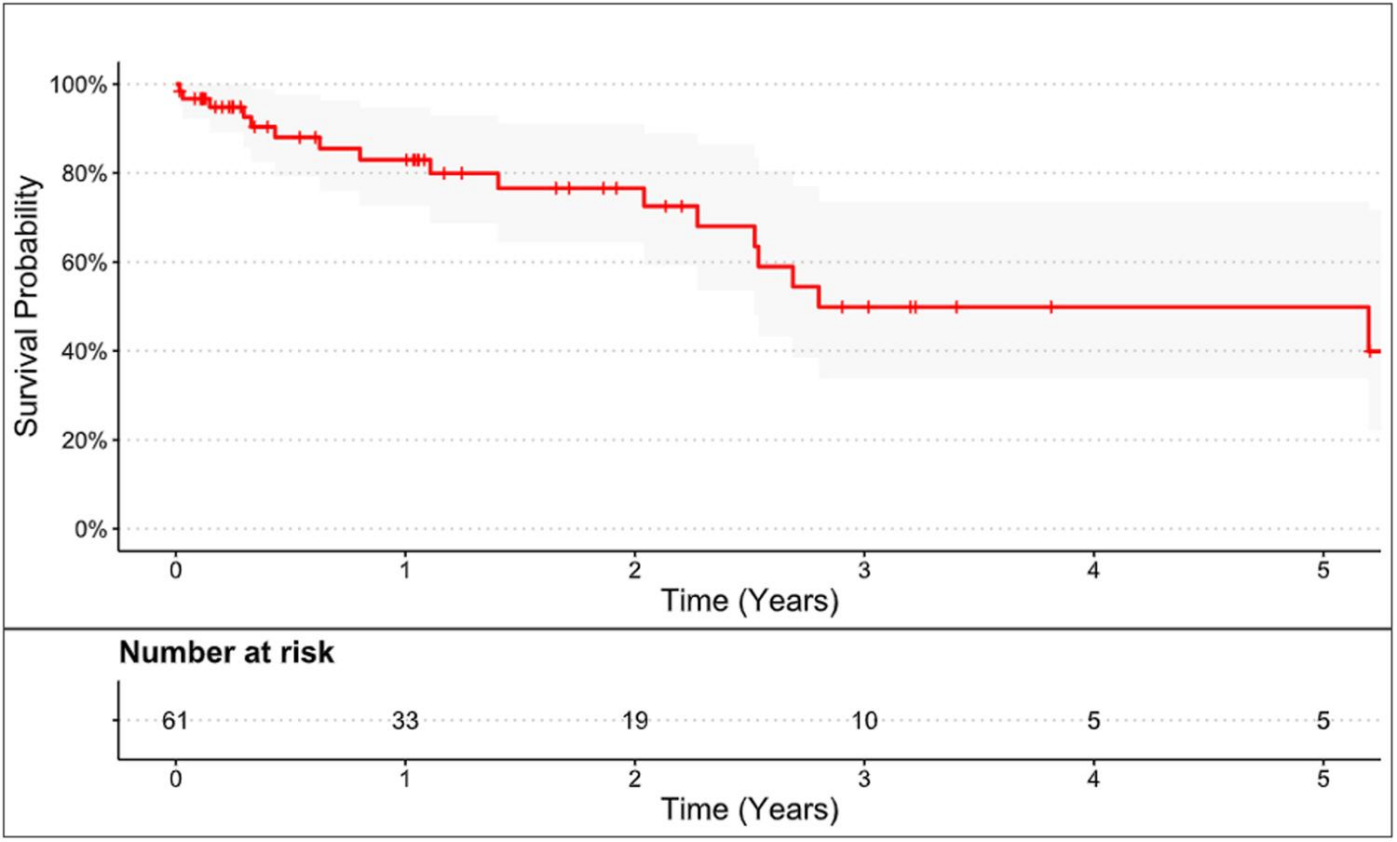
Kaplan-Meier Estimates for Early and Midterm Survival Following Cardiac Surgery After TAVR. The *red curve* represents survival, and the shaded region is the 95% CI. Patients at risk are listed below the graph.

The median duration of follow-up was 1.2 years (IQR, 0.3-3.2 years), during which 18 patients died. As shown in **Figure 1**, Kaplan-Meier 1- and 5-year survival estimates were 83% and 50%, respectively.

## DISCUSSION

In the early TAVR era, the procedure was primarily reserved for high- or prohibitive-risk patients, with subsequent cardiac surgery typically performed in emergent settings to address complications such as annular rupture, TAVR valve migration, and coronary occlusion. However, as TAVR indications expanded to include low- and intermediate-risk patients and younger individuals, there has been a notable increase in the frequency of subsequent cardiac operations. The key findings from this study show that the most common indications for cardiac surgery in patients requiring explantation of the TAVR prosthesis were structural TAVR valve deterioration (stenosis or regurgitation), non-structural valve deterioration (paravalvular leak), and prosthetic valve endocarditis. In contrast, among those who did not require TAVR valve explantation, MV disease, and coronary artery disease were the primary indications for surgery. Despite the high-risk nature of these operations, the operative mortality rate in this series was acceptably low at 6.6%, comparable to rates reported in similar single-centre studies.^5^^,^^8^^,^^9^^,^^11^

The cohort in this study consisted of predominantly elderly patients (median age 73 years) with a significant burden of comorbidities, including hypertension, dyslipidaemia, and diabetes mellitus—characteristics that align with those typically seen in the TAVR population. Notably, 48% of patients had previously undergone cardiac surgery, underlining the complexity of this population. At the time of surgery, the majority were symptomatic, with 77% exhibiting NYHA class III or IV symptoms. Despite the low-to-intermediate risk profiles, as indicated by preoperative STS-PROM scores, these patients faced significant morbidity, suggesting that the decision to proceed with cardiac surgery after TAVR requires careful consideration of both clinical and procedural risks, and also implies that the STS-PROM calculator may not appropriately capture the true risk associated with such complex operations.

The results from this study align with reports by Fukuhara et al^11^ and Jawitz et al,^8^ where TAVR valve deterioration and paravalvular leaks were also the primary indications for redo SAVR. Further, in the present study, nearly 50% of patients underwent cardiac surgery following TAVR due to progression of mitral or tricuspid valve diseases, and coronary artery disease. Notably, the median interval between the initial TAVR procedure and subsequent cardiac surgery was 19 months, suggesting that there may have been unrecognized or unaddressed associated valve pathology present at the time of the initial TAVR procedure. Previous studies have shown that residual MV regurgitation after TAVR is associated with increased 1-year mortality, and MV stenosis, which constitutes a significant coexisting valvular heart disease burden in patients with aortic stenosis undergoing aortic valve replacement, was found to be an independent predictor of adverse 1-year clinical outcomes, including mortality and heart failure-related hospitalizations.^14–16^ Similarly, moderate or severe residual tricuspid valve regurgitation after TAVR is associated with increased all-cause mortality.^17^ These findings highlight the importance of assessing nonaortic valve pathology in patients referred for TAVR.

In this study, aortic root enlargement or replacement was required in 33% of patients undergoing redo SAVR. Notably, the need for aortic root replacement did not differ significantly between patients with balloon-expandable versus self-expandable valves, suggesting that both valve types present similar challenges during explantation. These findings are consistent with the study by Bowdish et al, who reported that aortic root enlargement or replacement was necessary in 28.8% of cases following TAVR explantation and SAVR, with no significant differences between patients who had balloon-expandable vs self-expandable TAVR valves.

Prosthetic valve endocarditis, including TAVR valve endocarditis, is typically associated with high morbidity and mortality.^18^^,^^19^ In our study, we observed no operative deaths among patients who underwent cardiac surgery for TAVR valve endocarditis, which contrasts with similar studies where operative mortality ranged from 3% to 25%.^5^^,^^8^^,^^9^ Importantly, the incidence of aortic root procedures was higher among patients who underwent redo SAVR for endocarditis than those without endocarditis (58.3% vs 19.0%). These findings align with the understanding that endocarditis often leads to more complex aortic valve and root involvement, necessitating more extensive surgical management.

### Limitations

This study is limited by its retrospective design and relatively small sample size. As a single-centre report, the findings may not be generalizable to all institutions, particularly as the complexity of cardiac operations in our cohort may be influenced by the tertiary referral pattern at our centre. Further, the surgical follow-up is limited, as most cardiac operations were performed in the last 3 years, restricting our ability to assess long-term outcomes. Also, data on patients’ clinical status at the time of their initial TAVR procedure were incomplete, which may limit our understanding of preoperative risk factors and early complications. Despite these limitations, this study provides granular and valuable data that addresses important gaps in our understanding of the indications for, and extent of, surgical intervention after TAVR and associated outcomes.

## CONCLUSIONS

This study suggests that cardiac operations following TAVR can be performed with an acceptable operative mortality, despite the STS-PROM calculator not fully capturing the unique risks associated with such complex operations. The most common indications for TAVR explant were structural valve deterioration (stenosis/regurgitation), paravalvular leaks, and endocarditis. Importantly, in patients who did not require TAVR explant, progression of MV and coronary artery disease emerged as the leading indications for subsequent cardiac surgery. These findings reinforce the need to consider the potential for nonaortic valve pathologies when planning TAVR, particularly as the procedure expands to include younger and low- to intermediate-risk populations.

## Data Availability

The data underlying this article will be shared on reasonable request to the corresponding author.

## ABBREVIATIONS

CABG: coronary artery bypass grafting
IQR: interquartile range
LVEF: left ventricular ejection fraction
NYHA: New York Heart Association
PASP: pulmonary artery systolic pressure
SAVR: surgical aortic valve replacement
STS-PROM: Society for Thoracic Surgeons-Predicted Risk of Mortality
TAVR: transcatheter aortic valve replacement
